# Using phenomics to identify and integrate traits of interest for better-performing common beans: A validation study on an interspecific hybrid and its Acutifolii parents

**DOI:** 10.3389/fpls.2022.1008666

**Published:** 2022-12-08

**Authors:** Diego Felipe Conejo Rodriguez, Milan Oldřich Urban, Marcela Santaella, Javier Mauricio Gereda, Aquiles Darghan Contreras, Peter Wenzl

**Affiliations:** ^1^ Genetic Resources Program, International Center for Tropical Agriculture (CIAT), Recta Cali-Palmira, Valle del Cauca, Colombia; ^2^ Bean Physiology and Breeding Program, International Center for Tropical Agriculture, Recta Cali-Palmira, Valle del Cauca, Colombia; ^3^ Department of Agronomy, Faculty of Agricultural Sciences, Universidad Nacional de Colombia, Bogotá, Colombia

**Keywords:** phenomic descriptors, phenomic proportions, interspecific hybrid, image analysis, machine learning

## Abstract

**Introduction:**

Evaluations of interspecific hybrids are limited, as classical genebank accession descriptors are semi-subjective, have qualitative traits and show complications when evaluating intermediate accessions. However, descriptors can be quantified using recognized phenomic traits. This digitalization can identify phenomic traits which correspond to the percentage of parental descriptors remaining expressed/visible/measurable in the particular interspecific hybrid. In this study, a line of *P. vulgaris*, *P. acutifolius* and *P. parvifolius* accessions and their crosses were sown in the mesh house according to CIAT seed regeneration procedures.

**Methodology:**

Three accessions and one derived breeding line originating from their interspecific crosses were characterized and classified by selected phenomic descriptors using multivariate and machine learning techniques. The phenomic proportions of the interspecific hybrid (line INB 47) with respect to its three parent accessions were determined using a random forest and a respective confusion matrix.

**Results:**

The seed and pod morphometric traits, physiological behavior and yield performance were evaluated. In the classification of the accession, the phenomic descriptors with highest prediction force were Fm’, Fo’, Fs’, LTD, Chl, seed area, seed height, seed Major, seed MinFeret, seed Minor, pod AR, pod Feret, pod round, pod solidity, pod area, pod major, pod seed weight and pod weight. Physiological traits measured in the interspecific hybrid present 2.2% similarity with the *P. acutifolius* and 1% with the *P. parvifolius* accessions. In addition, in seed morphometric characteristics, the hybrid showed 4.5% similarity with the *P. acutifolius* accession.

**Conclusions:**

Here we were able to determine the phenomic proportions of individual parents in their interspecific hybrid accession. After some careful generalization the methodology can be used to: i) verify trait-of-interest transfer from *P. acutifolius* and *P. parvifolius* accessions into their hybrids; ii) confirm selected traits as “phenomic markers” which would allow conserving desired physiological traits of exotic parental accessions, without losing key seed characteristics from elite common bean accessions; and iii) propose a quantitative tool that helps genebank curators and breeders to make better-informed decisions based on quantitative analysis.

## Introduction

Genebank plant genetic resources comprise the representative diversity of genetic material contained in traditional varieties and modern cultivars, as well as in the crop wild relatives and other wild plant species that can be used now or in the future for food and agriculture (Wang and Zhang, 2011). Currently, there are about 1,750 genebanks worldwide that conserve 7.4 million accessions of agricultural genetic resources (Noriega et al., 2019). Eleven CGIAR genebanks conserve about 730,000 accessions among crops, trees, and forages, of which the International Center for Tropical Agriculture (CIAT) conserves 37,987 bean accessions, 23,140 forage accessions and nearly 6,000 Cassava accessions (Noriega et al., 2019). Despite this great diversity, only approximately 10% of the accessions from the 1,750 genebanks is used in plant breeding, mainly because of poor phenotypic and genotypic characterization or lack of agronomic traits evaluation (de Carvalho et al., 2013; Tadesse et al., 2019; Nguyen and Norton, 2020; Kholova et al., 2022).

*P. acutifolius* (tepary bean) is an important species in common bean breeding, due to its adaptation to abiotic and biotic stress (Singh and Munoz, 1999; Porch et al., 2013; Kusolwa et al., 2016). The use of cultivated and wild relatives of *P. vulgaris* by the common bean breeding program at CIAT started in the 1980s, with the aim of generating lines with elevated levels of introgression from *P. acutifolius* and/or *P. parvifolius ~ P. montanus* (Debouck, 2021), using techniques such as congruity backcrossing (CBC) and recurrent backcrossing (RBC) (Haghighi and Ascher, 1988; Mejía-Jiménez et al., 1994; Singh et al., 1998) with the help of bridge genotypes.

Classification of hybrids based on phenotypic traits was done in the 1960s (Allendorf et al., 2001), however, the detection of morphological traits usually assumes that hybrids are phenotypically intermediate to the parents. This is often not the case, because hybrids express a mosaic of parental phenotypes (Arnold, 1997) influenced also by environmental conditions. Furthermore, morphological characters do not allow determining whether an individual is a first-generation hybrid (F1), a backcross or late generation hybrid (Allendorf et al., 2001).

Recently, there have been published studies that promote characterization processes using phenomic descriptors. When compared with conventional descriptors, these showed a better capacity for analysis of phenotypic variability (Rosero et al., 2019; Nankar et al., 2020). Phenomic descriptors has both qualitative and quantitative characters and deal with agronomic, morphological, physiological, and colorimetric traits of accessions which are captured by proximal sensors such as cameras, fluorometers, trichromatic, multispectral and hyperspectral sensors. Phenomic descriptors have a “high-throughput” character of data, which means, hundreds of accessions can possibly be characterized/screened in a reasonable time. However, the sheer volume, variety and veracity of imagery and remote-sensing data still present limits in data analysis (Singh et al., 2016).

To solve this problem, there are currently several machine learning models, such as partial least squares (PLS), random forest (RF), support vector machines (SVM), and neural networks (NN) used in phenomics data analytics (Araus et al., 2022). Based on phenomic traits, classification of accessions and their hybrids has been attempted. This has been made possible with the development of phenomic and machine learning methods with thousands of data points from each individual, (Parmley et al., 2019; Soltis et al., 2020; Feldmann et al., 2020; Henao-Rojas et al., 2021).

In this work, we propose a Phaseolus-oriented methodology for the detection of phenomic proportions of interspecific hybrids with respect to their parents. We used multivariate and machine learning methods to characterize and classify three parental line accessions (a cultivated *P. vulgari*s, a domesticated *P. acutifolius*, and a wild *P. parvifolius* - P*. montanus*) with its interspecific hybrid accession.

The phenomic proportions correspond to the percentage of parental descriptors which remain expressed/visible/measurable in the particular interspecific hybrid. Correspondingly, in this study, phenomic proportions show the phenomic traits portions quantified in the three parental lines and verified in an interspecific hybrid. We hope this methodology will provide a first step to help genebank curators, breeders, physiologists and others to i) make detailed quantitative comparisons of selected phenomic traits between accessions of interest, and ii) better manage and understand their genetic resources. After some generalization using other parents/hybrid collections or random selection, this methodology could facilitate a deeper understanding about i) crossings, heritability and breeding success; ii) functional trait diversity; iii) species domestication/evolution and genetic recombination; and iv) how to substantially increase genetic gains (in tandem with genomics).

## Methodology

### Plant material and experimental design

Materials used in this study were *P. acutifolius* (G40001 – CIAT genebank accession number), *P. parvifolius - P. montanus* (G40102), *P. vulgaris* (G5773) and their interspecific cross hybrid line INB 47 (G52443), all obtained from the bean collection at CIAT Genebank. Accessions G40001 (*P. acutifolius*) and G40102 (*P. parvifolius - P. montanus*) display a type III growth habit (indeterminate prostrate growth); while accessions G5773 and hybrid G52443 exhibit a type I growth habit (determinate bush growth; growth habits defined according to Fernández de Córdoba et al., 1991). Accession G40001 showed heat and drought resistance (Mejía-Jiménez et al., 1994) and G40102 was highly resistant to common bacterial blight (CBB) (Singh et al., 1998).

The interspecific hybridization was a product of artificial crossings, carried out by the CIAT common bean breeding program, from crosses between a popular commercial bean variety Ica Pijao, *P. parvifolius* (G40102) and an interspecific line, with five cycles of congruity backcrossing (CBC_5_) between Ica Pijao and *P. acutifolius* (G40001) (Mejía-Jiménez et al., 1994). The pedigree of the interspecific hybrid line is as follows: INB 47 (G52443) = ICA PIJAO x (G40102 x (ICA PIJAO x (G40102 x (ICA PIJAO x (ICA PIJAO x (ICA PIJAO x G40001; CBC_5_).

**Figure 1:**
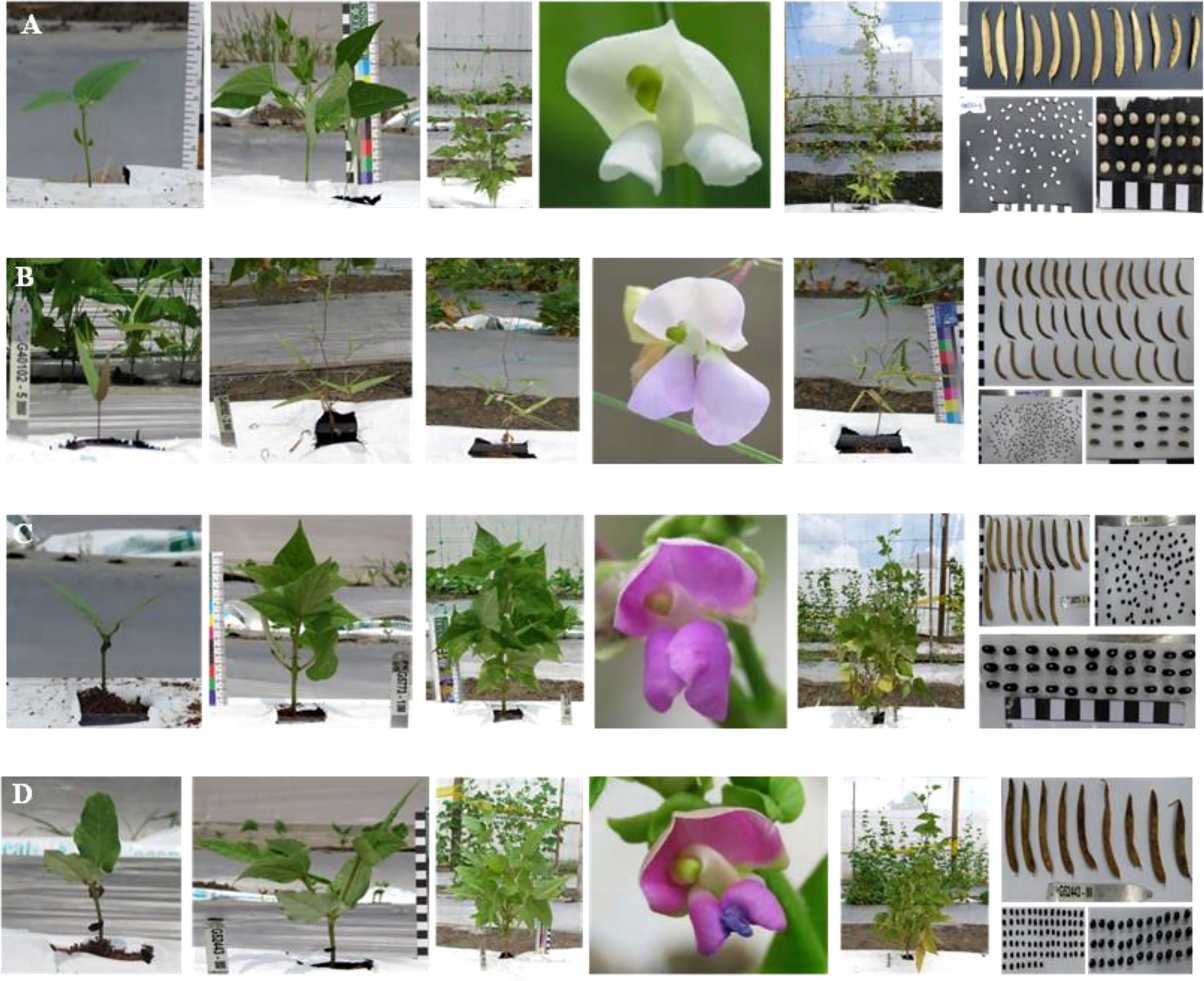
Phenotypic characteristics of the *Phaseolus* lines and interspecific hybrid, from its initial stages until harvest. **(A)** *P. acutifolius* (G40001), **(B)** *P. parvifolius* (G40102), **(C)** *P. vulgaris* - ICA Pijao (G5773) and **(D)** Interspecific hybrid (G52443 - INB 47). The figure shows the variation of morphological traits of the common bean accessions evaluated.

The INB 47 line was developed from ten (10) cycles of selection pressure based on commercial characteristics including growth habit, seed type and yield in the experimental station in Santander de Quilichao and in Palmira (both sites in Colombia), CIAT (Personal communication, Common bean breeding program, CIAT). The selection was mainly focused on conserving the commercial seed type similar to *P. vulgaris –* Ica Pijao, while introducing resistance to bacterial diseases (Mejía-Jiménez et al., 1994). The phenotypic characteristics of the parental lines and the interspecific hybrid are shown in **Figure 1**. These characteristics were observed during the experiment at the regeneration station of CIAT’s genetic resources program (GRP) in Palmira.

Our experiments were performed in two periods: from October 2018 to January 2019, and from January 2019 to April 2019, in a mesh house at CIAT, Palmira, Colombia (3°30′17″ N, 76°21′24″ W, 950 masl). The cultivation protocol was used according to standard operating procedures used in CIAT genebanks when multiplying accessions. The experimental conditions inside the mesh house presented: i) an average daily temperature of 38°C, with a daily maximum temperature of 41°C at midday hours, and daily minimal temperature of 27°C; ii) a minimum relative humidity of 31% and a maximum of 65%; and with iii) an average photosynthetically active radiation (PAR) of 1680 μmol m-2 s-1 during the sampling of physiological variables (please, explore variables at: https://photosynq.org/projects/domestication-syndrome/explore); iv) 12 h of natural light, and v) conventional agronomic management and fertigation using drip irrigation. The plants were sown in a substrate of coconut fiber substrate for hydroponic systems (120 × 40 × 40 cm), composed of 100% peat, which is highly efficient in conserving water and guarantees health development in the early stages of cultivation (elaborated in Spain by the company Berger, https://www.berger.ca/en/horticultural-products/). Water irrigation and fertilization scenarios were the same for all plants (**Supplementary Material Table 1** - Fertilization).

Each plant was tutored with agricultural mesh, starting at germination, to minimize human intervention in the span of plant development and guarantee adequate growth. The experimental design was a complete randomized block design, in which each accession acted as a treatment and each plant as an experimental unit. Five (5) plants per accession were planted, and three (3) independent technical repetitions measured in each period. To evaluate differences between accessions with special focus on the hybrid, we decided to compare morphometric, physiological, and agronomic traits.

### Morphometric descriptors

We evaluated the morphometric aspects of pod shape and lateral seed shape. Seed morphological characterization was carried out using phenomic descriptors based on image analysis, including the following traits: seed Area (cm^2^), Perimeter (cm), Width (cm), Height (cm), Major, Minor, MinorFeret, MajorFeret, Aspect Radio (AR), Circularity, Roundness, and Solidity according to Schlautman et al. (2020) and Rosero et al. (2019) (see details in **Supplementary Material Table 2** – Morphometric descriptions).

The digital images of each of the accession’s pod/seed were obtained using Canon SX60 HS camera with a digital resolution of 16.1 megapixels, an image area of 1080 × 1080 pixels in an automatic format. We captured images in a RAW format to ensure maximum image quality. Images were taken separately for seeds and pods. Each picture was processed using the DCRAW plugin of ImageJ 5.4 (https://imagej.net/software/fiji/).

After plant harvest, images were captured from pods and seeds of every accession, considering the level of luminosity and the contrast of the background. To capture color of the seeds, we used a contrasting background and standardized color scale 24ColorCard Camera Trax Card-3x5 (**Supplementary Material Figure 1** – Color scale and photobox used). Images of the pod shape and lateral shape of the seeds were processed using ImageJ software (Eliceiri et al., 2012), following the protocol: (I) Settlement of the scale (pixels to cm), (II) Image binarization using the Max Entropy and Huang methods (Huang and Wang, 1995), (III) Definition of regions of interest (ROI) from pod and seed selections, and (IV) Extraction of morphometric measurements of each of the selected ROIs (**Supplementary Material Figure 2** – imagen analysis process).

### Physiological descriptors

For physiological measurements, we used the MultispeQ device (Kuhlgert et al., 2016; PhotosynQ, USA). The device includes climatic and plant variables that facilitate characterizing the physiological performance of plants in their environment. All data were captured at midday between 11:00 am and 1:00 pm, with the aim of comparing the data collected under more stable temperature and lower air humidity (RH) under mesh house conditions. Measurements were taken three times a week in three technical (plant as experimental unit) repetitions per accession in all three replicates and both periods. In total, 1,022 MultispeQ observations were captured for the four accessions. Samplings were carried out every 15 days from December 22, 2018 to April 23, 2019. Samples were taken during phenological stages 22 to 85 according to the BBCH scale from plant branching until harvest maturity (Feller et al., 1995). The classical protocol was used: Leaf Photosynthesis MultispeQ V1.0 (the raw data are available at: https://photosynq.org/projects/domestication-syndrome; ID 5685).

Briefly, the MultispeQ proximal sensors measure photosynthetic parameters including: i) quantum yield of photosystem II (ΦII – Phi2); ii) non-photochemical quenching (ΦNPQ - PhiNPQ); iii) energy losses for heat dissipation (ΦNO - PhiNO); iv) relative chlorophyll (Chl); v) linear electron flux (LEF); vi) leaf temperature differential (LTD); vii) maximum variable fluorescence at a steady-state conditions (Fm’); viii) minimum variable fluorescence during dark phase after a steady-state (Fo’); ix) variable fluorescence at a steady-state conditions (Fs’); x) efficiency of open reactions centers in the light (Fv’/Fm’); xi) fraction of open PSII centers when QA is oxidized (qL); xii) photochemical quenching relating PSII maximum efficiency (qP); xiii) fluorescence decrease ratio (RFd), and xiv) leaf thickness (Kuhlgert et al., 2016; Fernández-Calleja et al., 2020; Deva et al., 2020). In addition, the device also measures environmental conditions like light intensity (photosynthetically active radiation, PAR), air temperature and air humidity (see **Supplementary Material – Figure 3** for temperature range, humidity and PAR). The data acquired can be visualized on the PhotosynQ platform (i.e. exploratory analysis of the data). Thus, at air temperatures above 32°C, photosynthetic activity is restricted in common bean of determinate growth type (Beebe et al., 2011; Deva et al., 2020).

### Yield components

To calculate seed yield, ten pods were taken randomly from each of three similar plants per accession during the final harvest at BBCH 89. Seeds were pre-dried according to the Genetic Resources Program methodology (Salazar et al., 2020). Seed weight was measured with high precision scales with seed average humidity of 14%. The dry weight of seed at harvest (PSW), weight of pod with seed at harvest (PW), dry weight of pod without seed (pod walls) (VW), number of seeds (SN) and the harvest index at the pod level (PHI = ((Dry weight of seeds at harvest)/(Dry weight of whole pod at harvest))x100) were determined.

### Multivariable analysis and Phaseolus accessions classification

Initially, we performed an outlier detection test using the Dobin library of R software (Kandanaarachchi and Hyndman, 2021). Principal Component Analysis (PCA) was carried out first on the parents, and the Principal Components (PC) with the highest contribution to the explained variance were extracted in each characterization group for each variable (performed using the FactorExtra library of R; Kassambara and Mundt, 2020). Predictor analysis was performed by random forests (randomForest library of R; Liaw and Wirner, 2018). Classification was performed on 100 trees, using 70% of the data for tree training, and 30% of the data for validation. For evaluating the classification model prediction with “out of bag” accuracy (OOB accuracy). The OOB is an error estimation technique used to evaluate the accuracy of the random forest (Janitza and Hornung, 2018). The OOB estimates accuracy across all classes (values above 1 - 10% are estimated as high accuracy; Kennedy et al., 2015).

The evaluation metric and confusion matrix were determined to observe the phenomic proportions of parent classification for each characterization group. The descriptors selected for each characterization component are determined by mean decrease in accuracy and the gini decrease index as parameters for feature selection. Analyses were run in R using the library “randomForestExplainer” and “caret” library (Paluszynska, 2017).

### Phenomic proportions of the interspecific hybrid with respect to its parents

Initially, the contributions of the PCs from the classification of the parent lines were used for weighting the phenomic descriptors of the interspecific hybrid. Subsequently, the weighted phenomic descriptor values of both parents and the interspecific hybrid are standardized to values between 0 - 1. The phenomic proportions are determined from classifying parents and an interspecific hybrid using random forests. Phenomic descriptors of importance in the classification will be determined for each characterization component using the gini index and mean decrease accuracy. The prediction of the confusion matrix in the interspecific hybrid will be considered as the phenomic proportions that it presents with respect to each of its parents. A confusion matrix is typically created representing the summary of the number of correct and incorrect prediction results broken down by each parental line.

**Figure 2:**
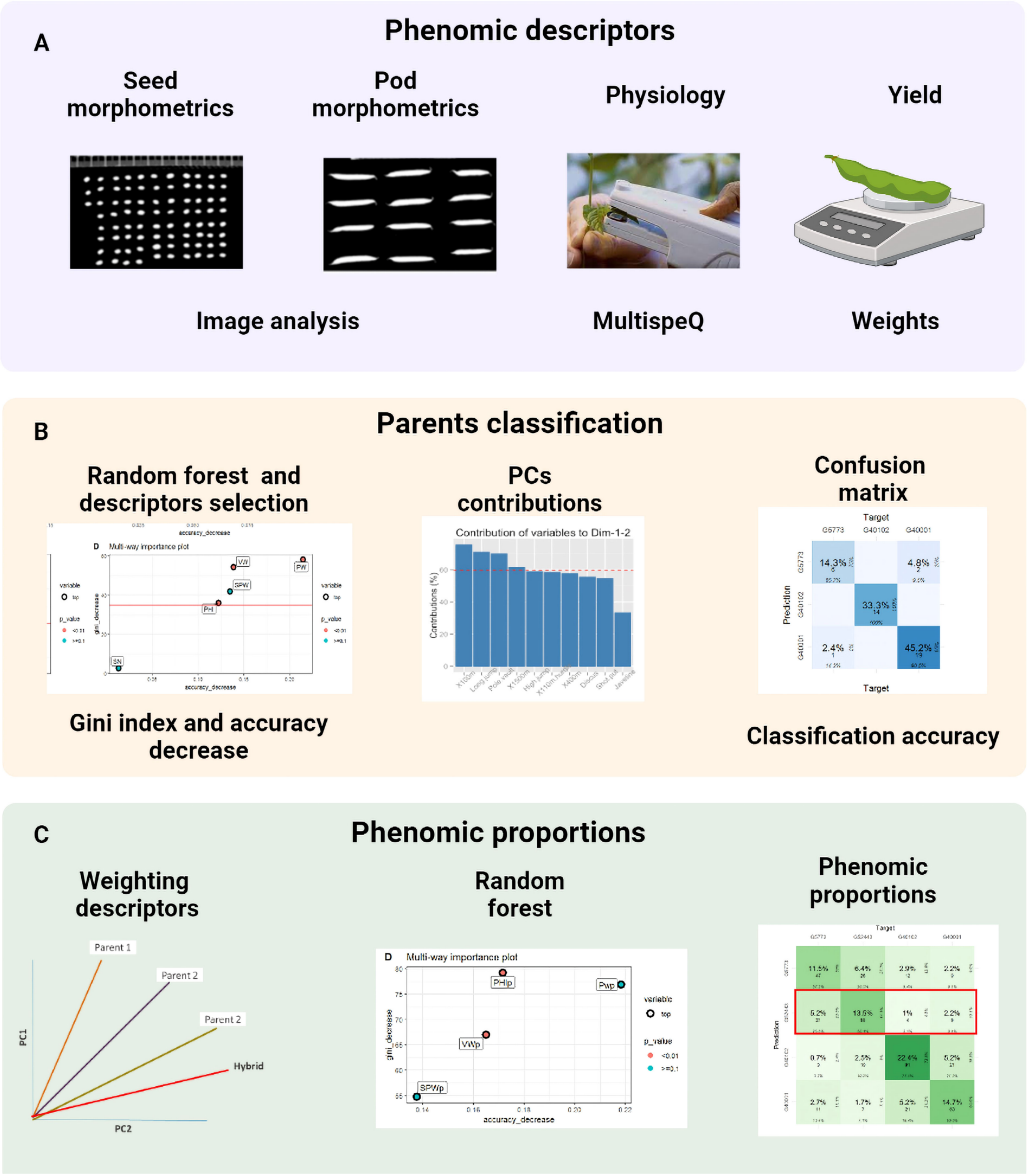
The procedure consists of three steps: **(A)** capture and processing of phenomic descriptors, **(B)** classification of parents using random forest and PCA and **(C)** phenomic ratios based on the weighting of the descriptors with the contribution of the PCs and phenomic ratios as the prediction of the hybrid with respect to its parents in the confusion table. This figure shows the different stages during the characterization process from data capture to data analysis.

**Figure 3:**
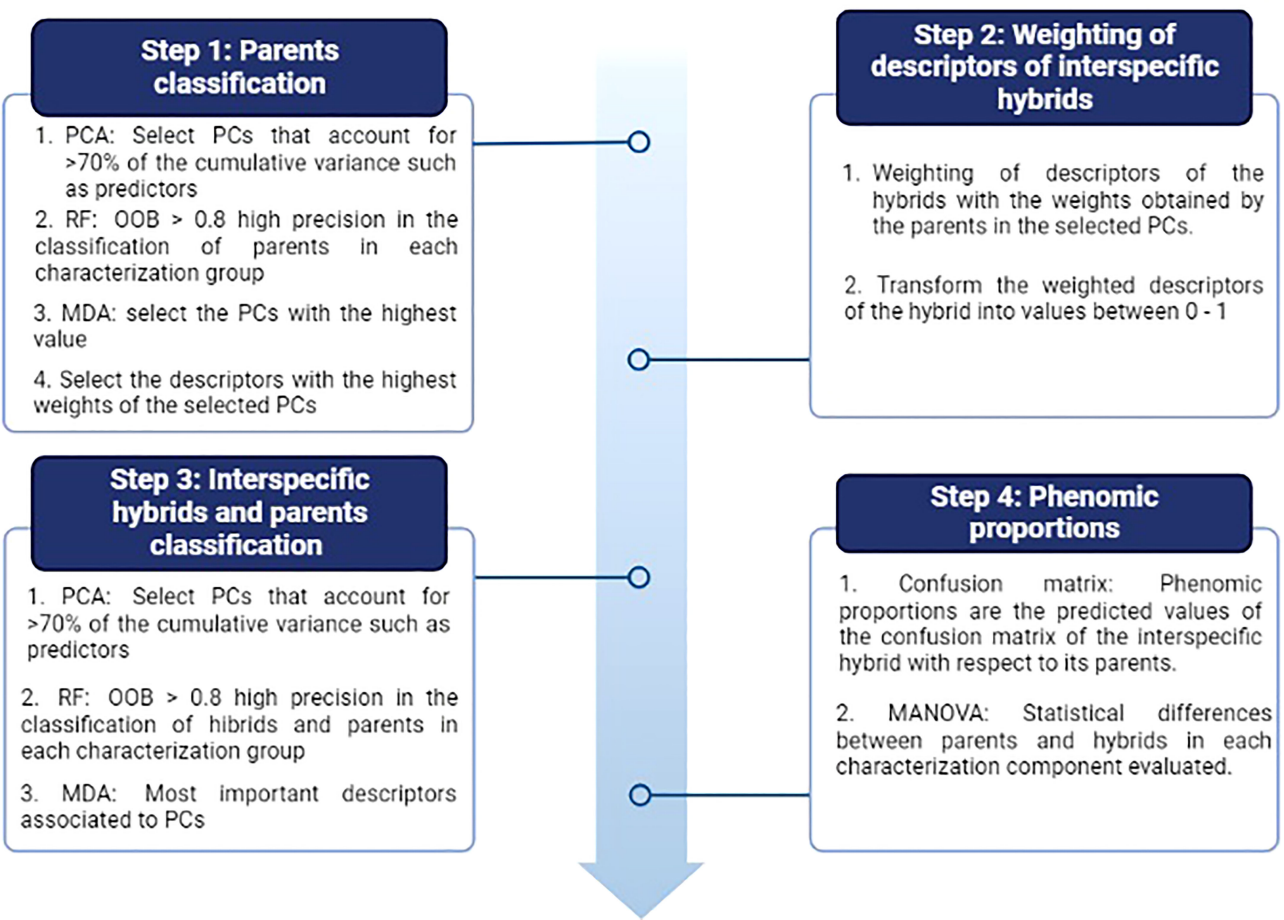
Procedure for the analysis of the phenomic proportions between interspecific hybrids and their parents. Four stages are observed that contemplate multivariate (PCA) and random forest (RF) analyses. OOB: Out of bag accuracy and MDA: Mean decrease accuracy. The figure shows the evaluation metrics used during each stage.

Finally, a non-parametric multivariate analysis of variance (MANOVA) was performed to determine if there are significant differences between the parents and their hybrids in each of the characterization components with the already prioritized descriptors. In our study we used MANOVA developed by Friedrich and Pauly (2018) which allows flexibility of normality assumptions and incorporates general heteroscedastic designs and potentially singular covariance matrices. It also improves the performance of small samples through bootstrap techniques. The analysis was performed using 10,000 iterations, modified ANOVA-type statistics (MATS), and the p-resampling value was determined from the parametric bootstrap approach (paramBS). MANOVA was performed for each characterization component separately (physiology, pod morphometry, seed morphometry and yield). In order to observe the significant differences between parents and its hybrid, the *post hoc* Tukey multivariate test was performed. This was done using the MANOVA.RM library of the free software R. The summary of the data analysis procedure can be seen in **Figures 2**, **3**.

## Results

The three parental accessions used in this study show contrasting phenotypic differences in morphological characteristics of the leaf shape, growth habit, flower shape and color, and seed size (**Figure 1**). *P. acutifolius* (G40001) (**Figure 1A**) presents an oval-lanceolate leaf shape and acute angles (less than 90 degrees) in conditions of high light intensity at midday hours. The flowers and seeds of *P. acutifolius* are white, with a straight pod shape, and a predominantly round-oval seed shape. *P. parvifolius* (G40102) (**Figure 1B**) is a wild accession with a lobed leaf shape, a light-purple flower color, and dark-purple and curved pods. *P. parvifolius* seeds are small, black to mottled grayish black, with a flattened truncated shape. *P. vulgaris* - Ica Pijao (G52443) (**Figure 1C**) has an oval leaf shape, dark purple flowers, mottled purple pods with slightly curved pod shape, and black seeds with a flattened oval shape. The interspecific hybrid (G52443 - INB 47) (**Figure 1D**) has a lanceolate leaf shape and acute leaf angles under high light intensity. It has purple flowers, with some flowers showing malformations in the floral wings. The pods are slightly curved with mottled purple colors, the seeds are black, and the seed shape is round cuboid.

The classification of the parent accessions using random forests, determined as predictive descriptors for seed shape were: Major, Area, Minot, Height and MinFeret; while in pod morphometry the predictive descriptors were: Major, Feret, Round, Aspect Radio (AR), Solidity and Area, presenting corresponding values of gini index higher than 40 and accuracy decrease 0.06 (**Supplementary Material Figures 4AS, 4CS**). The physiological variables, the phenomic descriptors Fo’, LTD, Fs’ and Fm’, and in the yield components VW (Valve weight), PW (Pod weight), SPW (Seed pod weight) and PHI (Pod harvest index), presented gini index values higher than 35 and accuracy decrease higher than 0.075 (**Supplementary Material Figures 4AS, 4CS**). The table of the contributions of the PCs to each of the predictive descriptors is shown in **Supplementary Material Table 3**. The classification of the parents can be seen in the confusion matrix (**Supplementary Material Figure 5** Matrix confusion parents’ accessions).

### Phenomic proportions of the hybrid respect to its parents

The low OOB value of 5.68% differences in the phenomic shape descriptors of seed of hybrid with respect to its parents. Interestingly, in the case of pod morphometric descriptors and physiological descriptors, the OOB values reached 22.44% and 36.76%, respectively; while 8.49% for the yield component (**Supplementary Material Table 4** - OOB error).

**Figure 4:**
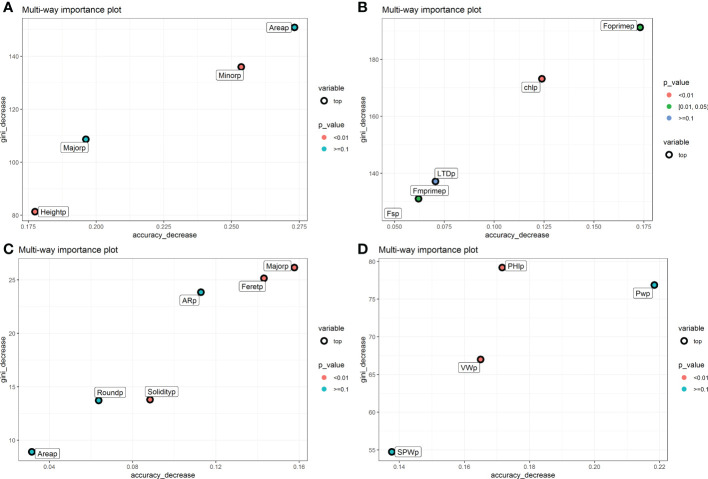
Gini index and accuracy decrease for feature selection in random forest for each characterization component in the hybrid. **(A)** Seed morphometric component, **(B)** Physiological component, **(C)** Pod morphometric component and **(D)** Yield component. The weighted phenomic descriptors are observed. The figure shows the phenomic descriptors of major importance in the classification of the parental accessions and the interspecific hybrid.

The phenomic descriptors of importance in the classification of the parental accessions and the hybrid are presented in **Figure 4**. Generally, the descriptors of seed morphometry, Area and Minor were the most important, while in pod morphometry the descriptors Major, Feret and AR presented accuracy higher than 0.250 (**Figures 4A, C**). In the physiological descriptors, Fm’ and Chl are the most important, and in the yield components the descriptors PHI and PW are the ones that presented the highest values of accuracy decrease with values higher than 0.125 (**Figures 4B, D**).

**Figure 5:**
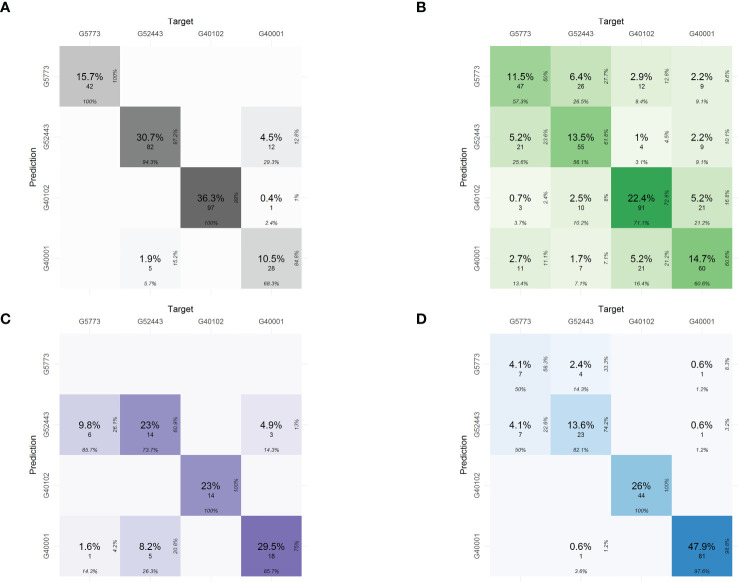
Phenomic proportions in the confusion matrices for classification of the interspecific hybrid in each of the characterization components using phenomics descriptors. **(A)** Seed morphometric component, **(B)** Physiological component, **(C)** Pod morphometric component and **(D)** Yield component. The confusion table shows the phenomic proportions of the interspecific hybrid. The phenomic proportions are the predictions of the hybrid with respect to the parental accessions.

Using the predictions in the confusion table, the phenomic proportions of each of the characterization components were determined (**Figure 5**). The relationships of the predictions of the hybrid with its parents are most closely related to the common bean parent *P. vulgaris* - Ica Pijao (G52443). The values of proportions are as follows: 5.2 % for physiological traits, 9.8% for pod morphometric traits, and 4.1% for yield components.

Despite the hybrid’s high relatedness to its *P. vulgaris* parent accession (the effect of multiple back-crosses), the hybrid presents phenomic proportions of 2.2% also with *P. acutifolius* (G40001) and 1% with *P. parvifolius* (G40102) accessions in physiological descriptors (**Figure 5B**), while 4.5% with *P. acutifolius* in seed morphometrics (**Figure 5A**), 0.6% for yield and 4.9% for pod morphometrics indicating successfully inherited traits from these parents as well (**Figure 5C**).

Phenomic proportions of the physiological components showed trait discrimination (difference) between the parents in traits like the Chl, Fm’, Fo’, LTD and Fs’, respectively. Furthermore (**Figure 4B**), it was observed that the parental *P. parvifolius* shows contrasting physiological behavior when compared with the other two parents. This can be explained by its wild origin and different morphometric characteristics. The interspecific hybrid is closely related to the *P. vulgaris* accession and also the *P. acutifolius* accession, indicating that it conserves physiological characteristics mainly from these two parents. However, the hybrid presents higher phenomic proportions of the *P. vulgaris* accession and not of the *P. acutifolius* accession (**Figure 5B**). For seed morphometry, the confusion matrix clearly separates the interspecific hybrid and the parents with high precision, showing the hybrid has unique characteristics in seed morphometry, despite sharing 4.5% of phenomic proportion with *P. acutifolius* (**Figure 5A**). In pod morphometry, it is clear that there is a difference of our hybrid regarding its *P. acutifolius* and *P. parvifolius* parental accessions (**Figure 5C**). This supports the fact that there is no clear trait separation between the *P. vulgaris* parental and the interspecific hybrid and again is likely an influence of multiple back-crossing and/or environment-based limitations of *P. acutifolius*-related traits.

The data after MANOVA fitting, supported the rejection of the statistical hypothesis associated with shared characteristics between the hybrid and its parents (**Table 1**). In the physiological characterization (MultispeQ data), there were no significant differences with the parentals *P. acutifolius* and *P. vulgaris* accession; while with *P. parvifolius* accession differences were highly significant (< 0.001) (**Table 1**). In pod morphometry, the interspecific hybrid showed no differences with *P. acutifolius* and *P. vulgaris* accessions. In seed morphometry, the hybrid showed no differences with the parental *P. vulgaris*, while in the yield components it showed no differences with *P. parvifolius* and *P. vulgaris* (**Table 1**) accessions. The MANOVA supports the statistical differences of the hybrid and its parents in each characterization component, contrasting with those obtained in the random forest analysis.

**Table 1:** MANOVA of parents and interspecific hybrid in each of the characterization components.

Characterization component	Accession	Contrast to:	p value	MATS	p - value Resampling
Physiological	*G40001* *(P. acutifolius)*	parvifolius	< 0.001	1016.229	< 0.001
		vulgaris	0.0073		
		hybrid	0.1053		
	*G40102* *(P. parvifolius)*	acutifolius	< 0.001		
		vulgaris	< 0.001		
		hybrid	< 0.001		
	*G5773* *(P. vulgaris)*	acutifolius	0.0073		
		parvifolius	< 0.001		
		hybrid	0.7523		
Pod morphometrics	*G40001* *(P. acutifolius)*	parvifolius	< 0.001	2538.441	< 0.001
		vulgaris	0.6099		
		hybrid	0.8856		
	*G40102* *(P. parvifolius)*	acutifolius	< 0.001		
		vulgaris	0.0004		
		hybrid	< 0.001		
	*G5773* *(P. vulgaris)*	acutifolius	0.6099		
		parvifolius	0.0004		
		hybrid	0.8301		
Seed morphometrics	*G40001* *(P. acutifolius)*	parvifolius	< 0.001	26348.12	< 0.001
		vulgaris	< 0.001		
		hybrid	< 0.001		
	*G40102* *(P. parvifolius)*	acutifolius	< 0.001		
		vulgaris	< 0.001		
		hybrid	< 0.001		
	*G5773* *(P. vulgaris)*	acutifolius	< 0.001		
		parvifolius	< 0.001		
		hybrid	0.6546		
Yield	*G40001* *(P. acutifolius)*	parvifolius	< 0.001	9697.0	< 0.001
		vulgaris	< 0.001		
		hybrid	< 0.001		
	*G40102* *(P. parvifolius)*	acutifolius	< 0.001		
		vulgaris	0.0302		
		hybrid	0.015		
	*G5773* *(P. vulgaris)*	acutifolius	< 0.001		
		parvifolius	0.0302		
		hybrid	0.9986		

The p-value for each parent and its relationship to the hybrid is observed.

In addition, it is observed that using seed morphometric descriptors, the interspecific hybrid shows differences from the three parents, indicating characteristics of the interspecific hybrid determined probably by the hybrid vigor.

## Discussion

High-throughput phenotyping methods can facilitate the use of genetic resources by estimating phenotypic traits of importance and identifying accessions of interest for pre-breeding and breeding programs (Nguyen and Norton, 2020). In this work, we explored phenomic descriptors that help discriminate selected *Phaseolus* accessions with their interspecific cross, using components based on physiological descriptors, seed and pod morphometrics and yield components.

Despite being crop relatives of *P. vulgaris*, Acutifolii species (*P. acutifolius* and *P. parvifolius*) have contrasting leaf, seed, and pod phenotypic characteristics (**Figure 1**). In addition, there are natural differences between domesticated and wild accessions (Mwale et al., 2020). The domestication syndrome of the Phaseolus genus is characterized by a reduction in pod shattering and increase in seed size, being these the most important traits in the adaptation of domesticated populations (Chacón-Sánchez, 2018). This explains the differentiation in our data for seed and pod morphometrics and reveals why they can serve as the most significant traits in quantification of phenomic proportions between the studied hybrid and its parents and under some generalization and verification can be used for a wider spectrum of hybrid evaluations.

Each of the accession’s classifications contained several phenomic descriptors that contributed to defining/identifying its uniqueness. In pod and seed morphometry, it is observed that the descriptors with highest contributions (**Figures 4A, C**), such as seed/pod Area, pod Feret, seed Height, seed MinFeret, seed Minor and seed/pod Major, are descriptors directly related to the organ (seeds or pod) size; while Solidity shows that the pod shape is influenced by its curvature and also by the shape of the seed. *P. parvifolius*, being a wild accession, does not present domestication syndromes (Chacón-Sánchez, 2018). This is evidenced by the pod and seed small size, being the primary discriminating descriptors in classifying the parvifolius accession. Both studied *P. acutifolius* and *P. vulgaris* have larger pod and seed sizes, likely due to the selection pressure of preferable domestication syndromes (Chacón-Sánchez, 2018). The domestication process directly influences pod and seed weights and can increase pod harvest index (PHI) (Rao et al., 2013). Interestingly, *P. acutifolius* generally has smaller seed size (Freytag and Debouck, 2002) but higher PHI compared to *P. vulgaris* (Rao et al., 2013). Higher PHI has strong heritability, is easily measured, and is related to drought resistance and low soil fertility tolerance. The *P. parvifolius* accession also lacks pod shape curvature, allowing simple visual differentiation and classification between accessions.

The physiological descriptors are extremely useful for accession classifications (**Figure 5B**), although less comparable to morphometric aspects. However, the lower weight of physiological traits is understandable when considering all physiological descriptors (in our case we measured photosynthetic/fluorescence traits and leaf-based data by MultispeQ) as greatly influenced by the environment, with possibly limited heritability and biologically relevant biochemical acclimation thresholds (resistance). The physiological traits inherently hold considerable genetic complexity, considering their crucial role in plant development and survival (Reynolds and Langridge, 2016). Alternatively, it is possible that *P. acutifolius* shows similarly reduced physiological behavior as *P. parvifolius* in the conditions where experiments were done. Although *P. acutifolius* and *P. parvifolius* are two different species (Buhrow, 1983; Schinkel and Gepts, 1989), *P. acutifolius* var. *latifolius* has been reported as an intermediate species between the domesticated *P. acutifolius* and wild *P. parvifolius.* This suggests that both studied accessions may share similar genetic background (Freytag and Debouck, 2002; Muñoz et al., 2006; Blair et al., 2012) and thus some physiological performance as mentioned above.

Regarding the physiological descriptors, it is clear that leaf components as Chl, Fo’, Fs’, Fm’, and LTD (**Supplementary Material Figure 4B**) present the highest contributions in the differentiation and classification between lines. Both *P. acutifolius* and its wild relative *P. parvifolius*, have similar physiological responses most likely due to the similar ecogeographic distribution of both species, associated with the arid areas of southern USA and northern Mexico (Freytag and Debouck, 2002). Similarly, the above-mentioned descriptors are closely related to photosynthetic efficiency and are recognized - in some scenarios - as indicators of abiotic stress resistance of individual accessions (Sánchez-Reinoso et al., 2019; Guidi et al., 2019). *P. vulgaris* usually exhibits higher sensitivity to drought stress compared to more resistant *P. acutifolius* (Rao et al., 2013; Polania et al., 2016). This can be closely related to the two independent domestication processes of *P. vulgaris* (Chacón et al., 2005), mainly influenced by differences in air/soil humidity and contrasting temperatures between the Mesoamerican and Andean races, presenting differences also in their photosynthetic adaptations (Lynch et al., 1992; González et al., 1995).

The studied interspecific hybrid line INB 47 is a product of interspecific crossings carried out by the CIAT common bean-breeding program. The selection process focused on obtaining adequate seed type, growth habit and yield characteristics from the parent *P. vulgaris.* No surprise then, which the studied interspecific hybrid presented low phenomic proportions with the *P. parvifolius* and *P. acutifolius* parent accessions. This is because agronomically-valued traits likely do not coincide with those two parental accessions. This is probably mainly because physiological traits were selected indirectly (in contrast to agronomically-important descriptors), with no apparent interest/knowledge in/of physiological traits at the time of selection by the breeders (Mejía-Jiménez et al., 1994).

Mejía-Jiménez et al. (1994) developed a group of CBC_5_ interspecific hybrids with *P. acutifolius*. These authors generated populations with high genetic frequencies of *P. acutifolius*, showing average introgressions of 8% in CBC_5_ using amplified fragment length polymorphisms (AFLP). Considering that the interspecific hybridization used in our study employed CBC_5_ crossed twice with the parents *P. vulgaris* and *P. parvifolius*, and that it was selected during ten selfing cycles, it is likely that introgression of the *P. acutifolius* has decreased. Nevertheless, the 2.2% of the phenomic proportions of *P. acutifolius* - predicted from the physiological characterization - evidence the successful introgressions from this parental line. In addition, the studied interspecific hybrid also preserves morphological traits similar to *P. acutifolius*, such as the lanceolate leaf shape and acute leaf angle at high light intensities (**Figure 1**). It could be interesting to evaluate the effect of these morphological traits on the abiotic stress resistance of this or other hybrids (after the methodology generalization).

Moreover, the studied interspecific hybrid keeps some characteristics that can influence the acclimatization process during abiotic stresses (Sánchez-Reinoso et al., 2019). This argues strongly in favor of conserving and characterizing accessions with intermediate phenomic proportions and could allow better understanding and quantifying (based on their GxE base) of the inherited traits and their proportions. It also would support accelerating genetic advances during more effective selection processes based on more newly available data types (semi-automatic remote sensing collection of data of highest interest).

Additionally, in hybrids, phenomic descriptors with the highest distinction powers (discrimination) could allow conserving desired physiological traits of *P. acutifolius* or *P. parvifolius* accessions, without losing key seed characteristics (e.g. size, color or taste) from their *P. vulgaris* parental line. Targeted accession evaluations can be performed by continuous monitoring even during the selection process (starting in already in the F1 generation). This would be based on the suggested machine learning techniques and selected traits of special interest for validating the functional introgressions of desired traits from crop wild relatives.

Our results demonstrate that the use of phenomic descriptors and machine learning analyses offer a very useful alternative for classifying hybrids, by using useful phenotypic and morphometric traits (with some degree of generalization and verification). In reality, breeders focusing on interspecific crossings should consider physiological and morphological traits identified in this study as part of an effective screening strategy. This would be especially true where some of these traits were to prove functional in certain environments (willow leaves, leaf angle, growth habit, PHI) or be connected to farmers preferences (seed color and size, pod shape etc.). Breeders would then be able to use other selection criteria apart from the laborious final yield components and seed type characteristics, and thus quickly estimate the introgression efficiency of functional traits in the progenies.

Currently, the CIAT genebank conserves 18 interspecific hybrid lines of *P. vulgaris* x *P. acutifolius* x *P. parvifolius* and 6 interspecific hybrid lines of *P. vulgaris* x *P. acutifolius*, which were selected based on the phenotypic traits conserving characteristics associated with its crop relatives. In addition, the CIAT genebank stores 326 accessions of *P. acutifolius*, including cultivated lines, landraces and wild accessions. However, only a fraction of the whole collection has been studied and characterized for key agronomic and physiological traits, heavily limiting their utilization in pre-breeding or breeding programs (Mwale et al., 2020). This suggests the urgent need to conduct experiments to explore phenomic traits of the *P. acutifolius* collection, including the genetically diverse wild tepary bean accessions as these offer a unique opportunity to find desirable genes with potential for introducing them into the genetic background of the domesticated tepary bean (Mhlaba et al., 2018; Mwale et al., 2020). Breeders may then be encouraged to start working within the acutifolius group. We believe that selected phenomic descriptors can also help identify suitable “bridge” genotypes for crossings between secondary and tertiary genepools and common beans. The development of phenomic markers (new phenomic proportions recognized as important descriptors) will contribute to germplasm management in genebanks as well (Nguyen et al., 2020). Selected and recognized phenomic descriptors will facilitate the detection of accessions with similar phenomic proportions, determining accessions with high phenomic redundancy, and likely helping germplasm curators even to effectively find duplicate accessions.

Our study demonstrates that selected phenomic descriptors’ data processed by a machine learning approach have the potential to discriminate between parental accessions or our studied hybrid. After some generalization (trait verification on different hybrid systems), this methodological approach may help breeders quantify any trait-of-interest introgression directly from different genepools or wild relatives increasing the chances of identifying important consumer target traits in elite common bean lines. After generalization, this methodology also will be able to identify hybrids with hybrid vigor due to the performance of unique phenomic traits. In addition, genome-associated phenomic markers could further contribute to the detection of genes of deep agronomic interest under abiotic and biotic stress conditions (Pasala and Pandey, 2020; Al-Tamimi et al., 2021; Dwivedi et al., 2020; Rassizadeh et al., 2021).

Detailed characterization of CIAT genebank conserved interspecific hybrids or new early breeding materials will likely show new traits with physiological or agronomic potential. In our study, the most contrasting characterization components with the highest precision and stability of the selected *Phaseolus* taxonomy classification are the seed and pod morphometry data.

This study was never intended as the end of classic crop descriptors used by genebanks curators. In reality, classic descriptors will always offer their unique potential. However, some of them can still be rather subjective, are often only qualitative, and require laborious effort to apply them. We have tried to build on the understanding and precision of such classic mostly qualitative descriptors by digitization of some of the crop responses, so as to use a quantitative approach to make some descriptors available to modern breeders and with potential selection power (QTL, GWAS, genomic selection etc.).

In our study we were able to evaluate selected phenomic traits and their ability to become “phenomic markers” and then establish digital descriptors. We also identified machine learning techniques, which allow us to differentiate between studied Phaseolus accessions and determine the similarities or differences of an interspecific hybrid with respect to its parental lines. In our experiment we performed the analysis with random forests, however the strategy can use various machine learning algorithms (Parmley et al., 2019), since the purpose is to determine the most important descriptors that discriminate generally between the parents and its hybrid. There are several algorithms that can have greater accuracy in the classification according to the needs of the researcher and the dimensionality of the phenomic data.

## Conclusions

In our work we demonstrate the use of phenomics and machine learning approach as analytical tools in understanding the phenotypic variability of selected Phaseolus accessions and quantifying the crossing effectivity in its related hybrid.

In our study, we quantified the physiological, morphological and yield proportional relatedness of parental lines with its hybrid, finding differences between all groups. Results indicate that the interspecific hybrid preserve intermediate yield characteristics from *P. vulgaris* and *P. acutifolius* parents; although, it has closer phenotypic proportions with *P. vulgaris* (6%). The phenomic proportions method can be a useful tool for the analysis of the closeness of lines/hybrids to their parents even by using traits with clear agronomic potential. However, also physiological data (MultispeQ) showed high potential for lines discrimination, especially towards the studied line of *P. parvifolius*. This complex of traits needs to be further studied and amplified in a wide range of genotypes to verify its value across species, genepools and environments. Our finding provides conclusive evidence that the integration of machine learning, classification algorithms and phenotyping tools promise to automate the precise quantification of phenomics proportion of parents in their hybrids.

## Data availability statement

The raw data supporting the conclusions of this article will be made available by the authors, without undue reservation.

## Author contributions

DC: Substantial contributions to the conception or design of the work; or the acquisition, analysis, or interpretation of data for the work. MU: Drafting the work or revising it critically for important intellectual content. MS: Drafting the work or revising it critically for important intellectual content. JG: Drafting the work or revising it critically for important intellectual content. AC: Analysis, or interpretation of data for the work. PW: Provide approval for publication of the content. All authors contributed to the article and approved the submitted version.
