# Supplementary figures and images for: Using phenomics to identify and integrate traits of interest for better-performing common beans: A validation study on an interspecific hybrid and its Acutifolii parents
Source: Front Plant Sci. 2022 Dec 8;13:1008666. doi: 10.3389/fpls.2022.1008666 (PMC9773562; doi:10.3389/fpls.2022.1008666)

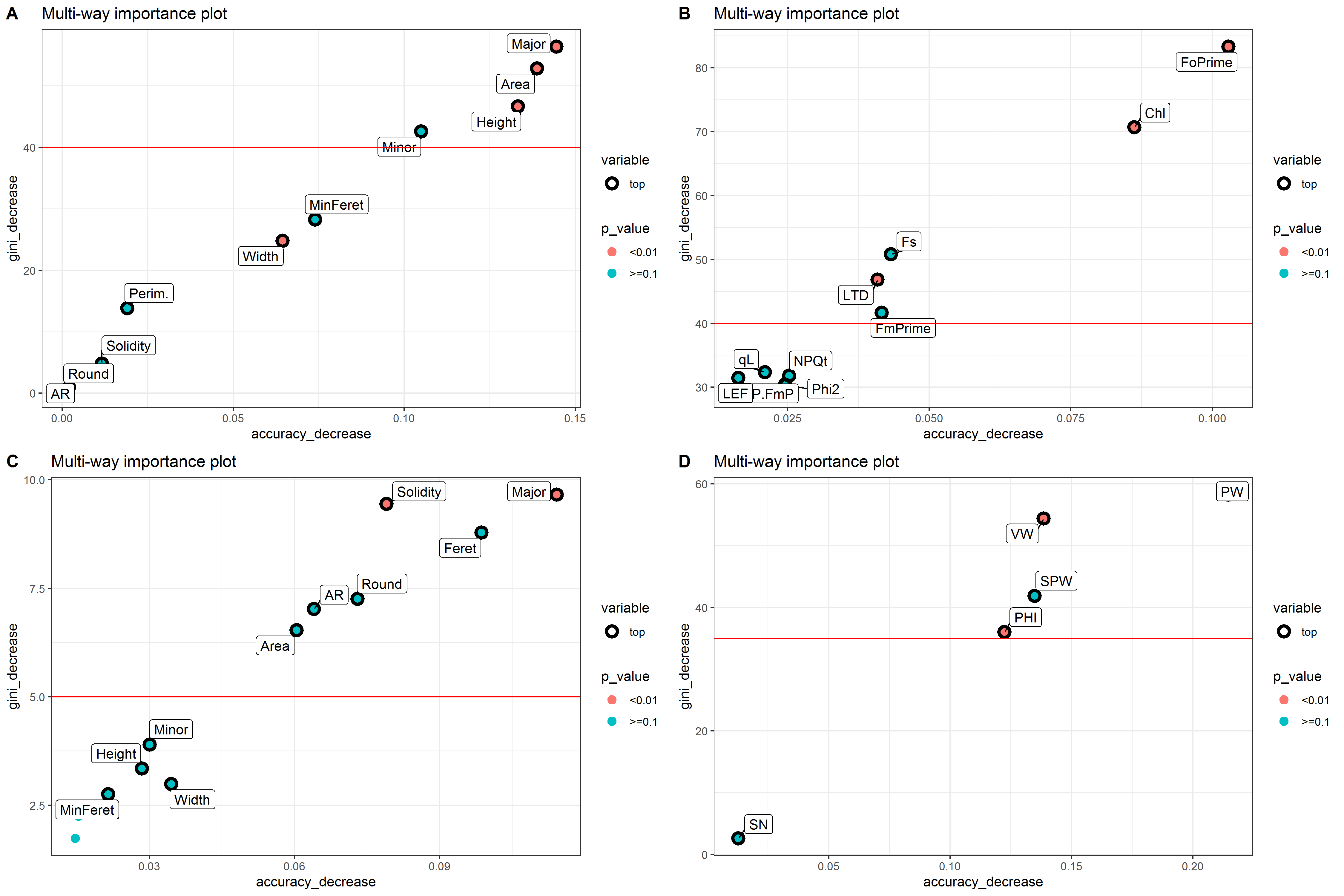

Supplement: Supplementary file 1 [file Image_1.jpeg]

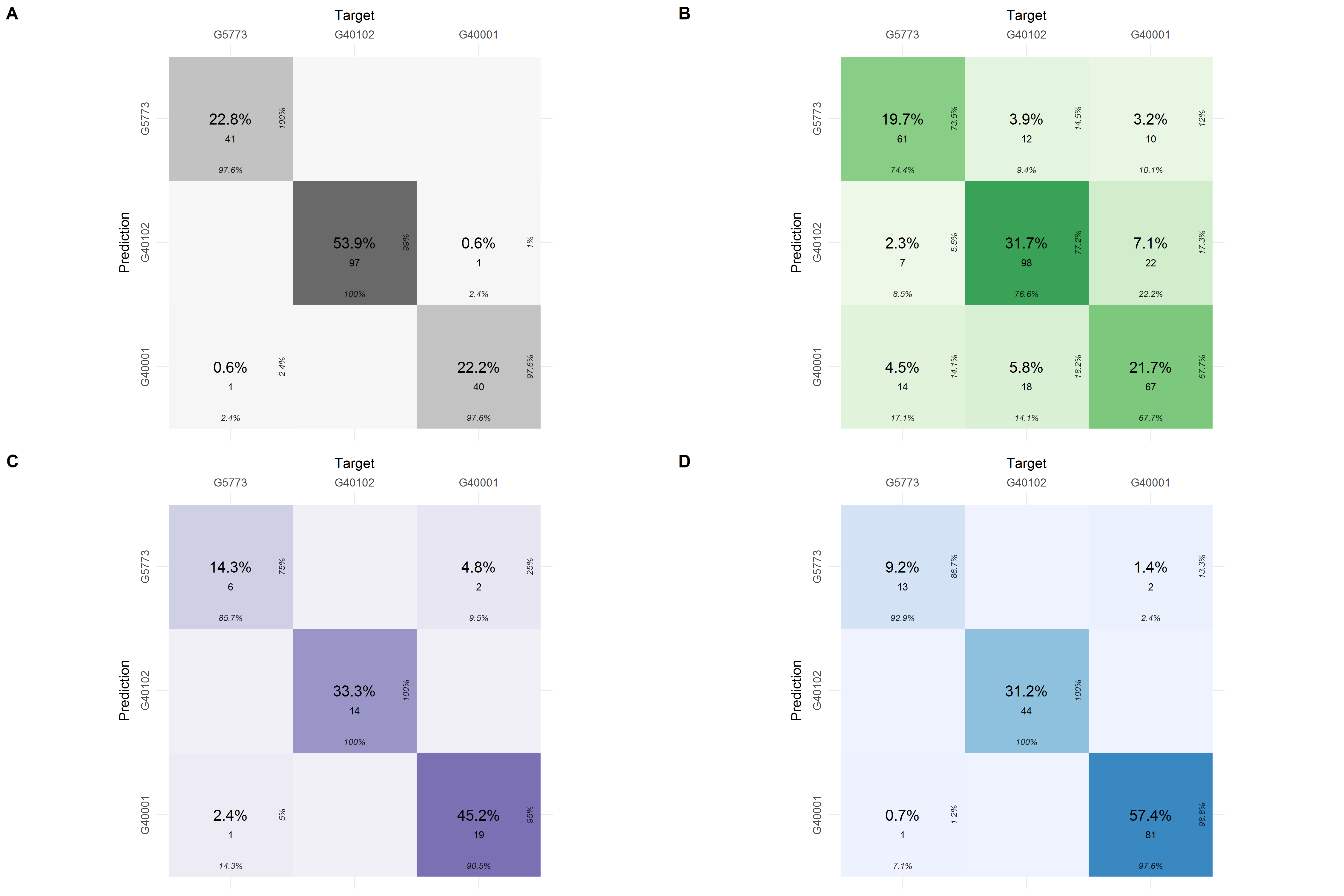

Supplement: Supplementary file 2 [file Image_2.jpeg]

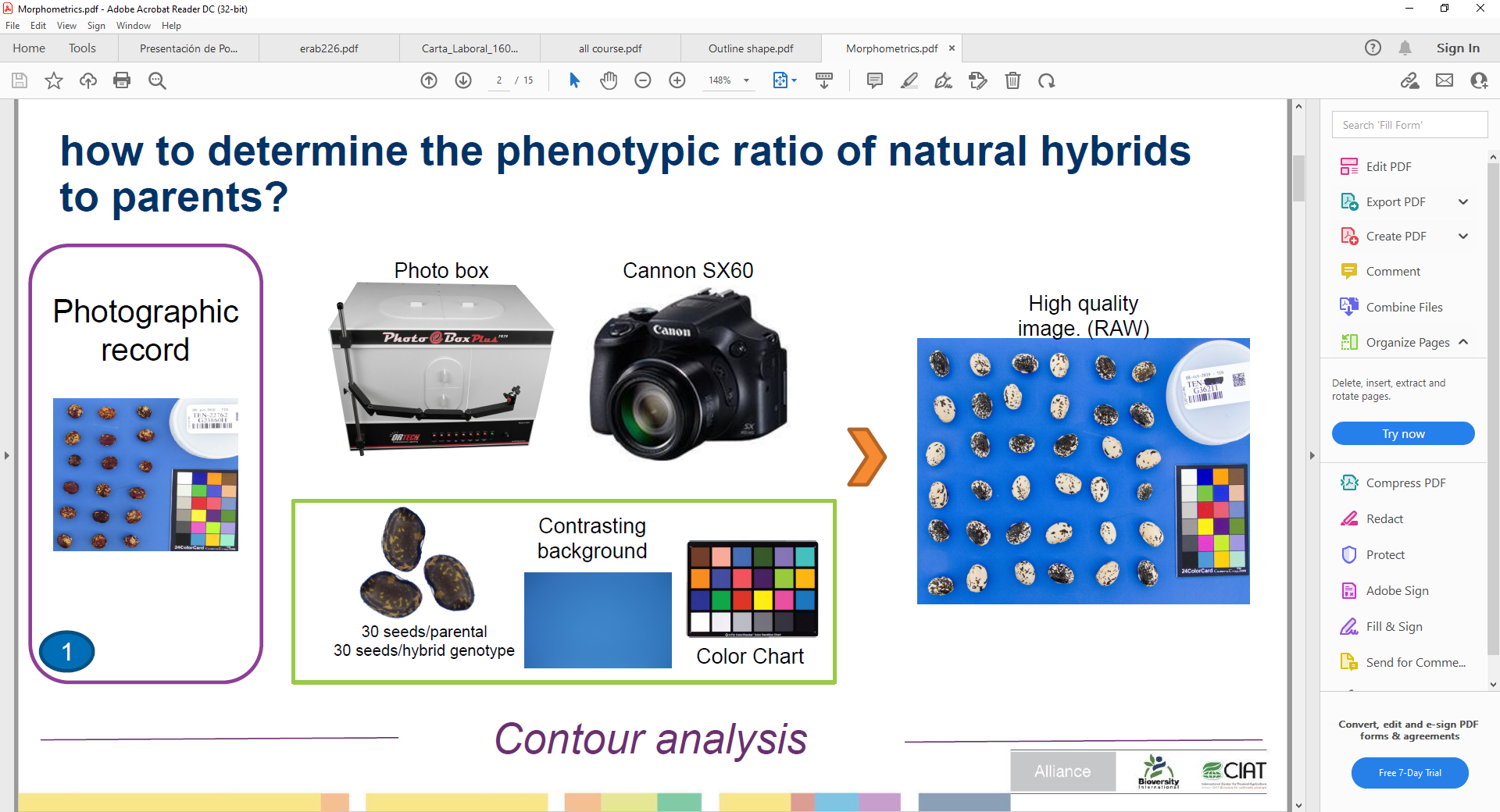


**Figure1S.** Seed and pod photographic station (PhotoBox), used camera and color card.

Supplement: Supplementary file 4 [file DataSheet_1.docx]

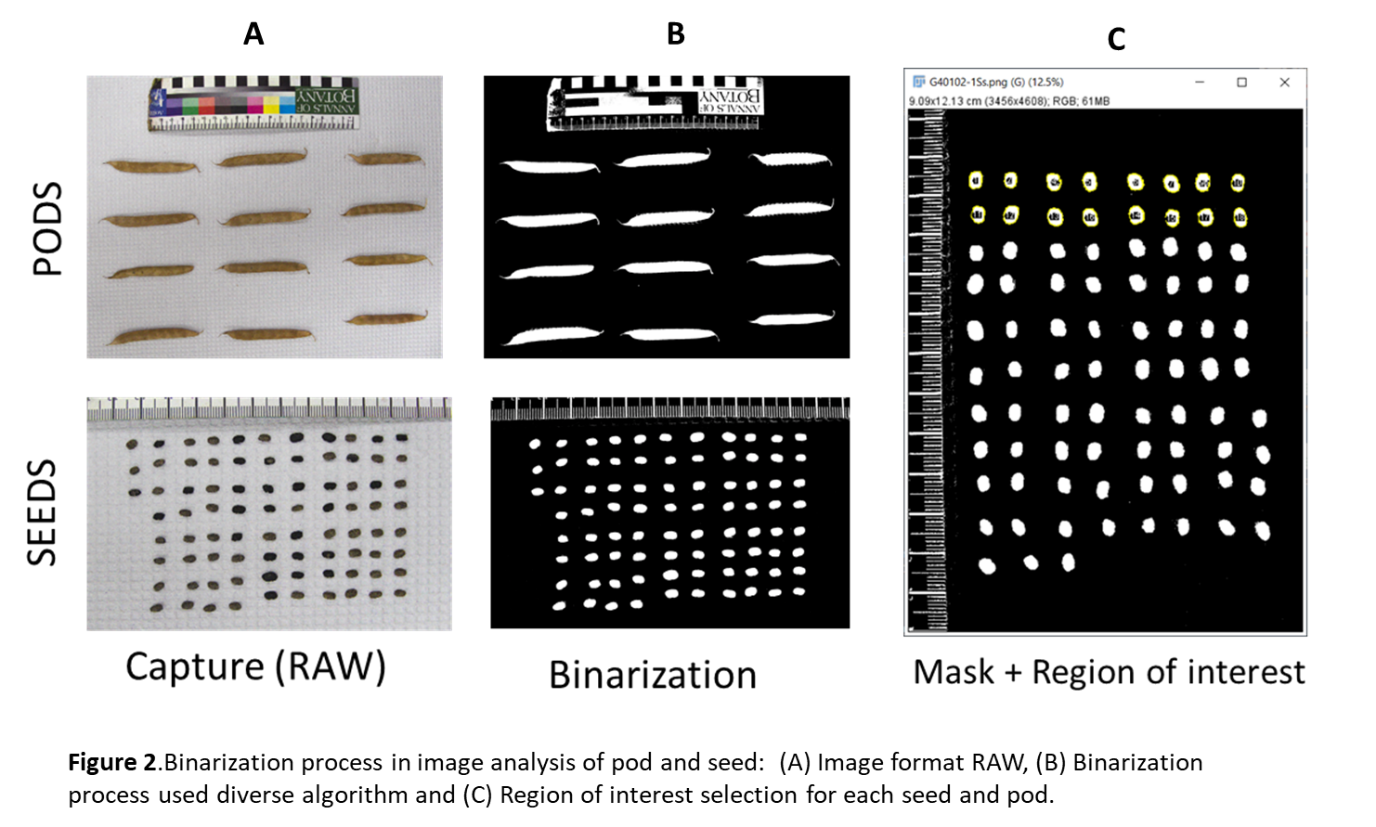


**Figure2S.** Image analysis process for the extraction of morphometric descriptors

Supplement: Supplementary file 5 [file DataSheet_2.docx]
