# Supplementary material for: Using phenomics to identify and integrate traits of interest for better-performing common beans: A validation study on an interspecific hybrid and its Acutifolii parents
Source: Front Plant Sci. 2022 Dec 8;13:1008666. doi: 10.3389/fpls.2022.1008666 (PMC9773562; doi:10.3389/fpls.2022.1008666)
Supplement: Supplementary file 6 [file DataSheet_3.docx]

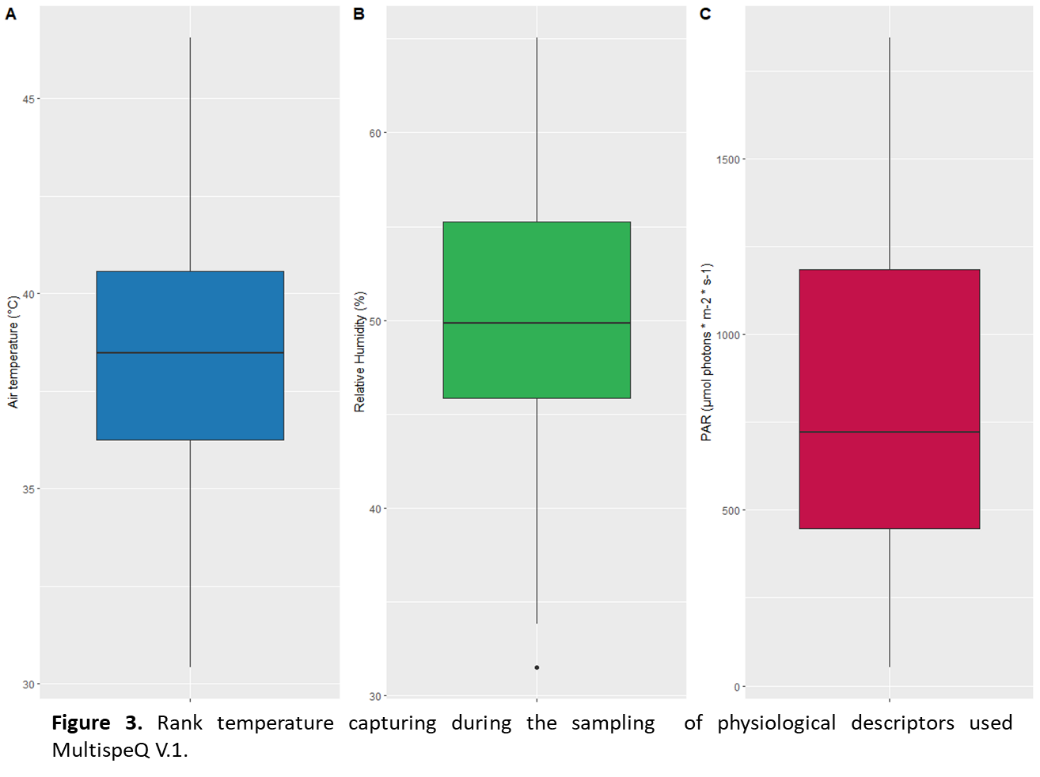


**Figure3S.** Ranges of air temperature, relative humidity, and photosynthetically active radiation during physiological samples
